# Subclinical hypothyroidism in population of aging Polish women and men over 65yrs old and cardiovascular risk factor, endogenous vitamin D levels and its gene receptor polymorphisms – PolSenior Study

**DOI:** 10.1186/1756-6614-6-S2-A44

**Published:** 2013-04-05

**Authors:** Andrzej Milewicz, Ewa Bar-Andziak, Łukasz Łaczmański, Anna Arkowska, Urszula Mieszczanowicz, Barbara Krzyżanowska-Świniarska

**Affiliations:** 1 Department of Endocrinology and Diabetology, Wroclaw Medical University, Wroclaw, Poland; 2 Department of Internal Medicine and Endocrinology, Medical University of Warsaw, Poland; 3 Department of Hypertension and Internal Medicine, Pomeranian Medical University, Szczecin, Poland

## 

Clinical consequences of subclinical hypothyroidism (SH) are still controversial, especially in aging persons. Recently the pleiotropic action of vit. D, also the influence on immune system, was shown and discussed. On the basis of randomly selected 723 women and men (382 men) over 65 yrs old from PolSenior Study we developed the frequency of SH in Polish aging population, and we compared the metabolic profile of cases with SH in comparison to healthy controls according to cardiovascular risk factors (CRF). The serum vit. D and its receptor (VDR) gene polymorphisms Fok-I and Bsm-I in both these groups were estimated. The elevated serum TSH level (over 4.5 mU/ml) with FT4 range: 10.3-25.7 pmol/l and elevated anti-TPO over 20.0 mU/ml showed 7.9% of women and 4.4% of men. The CRF profile: waist circumference, BMI, serum total cholesterol, HDL and LDL cholesterol, triglycerides, fasting glucose, insulin and HOMA were comparable in both groups. Also the endogenous vit. D serum levels were comparable between the group of patients with elevated anti-TPO and elevated or decreased TSH in comparison to controls with euthyroidism. However the analysis of Fok-I and Bsm-I polymorphisms of VDR and thyroid immune status showed higher frequency of recessive genotype bb in person with elevated TSH and anti-TPO (p=0.0334). Conclusions: aging people with subclinical hypothyroidism don’t present higher risk for CVD. The relation between vit. D and its receptor gene polymorphisms and thyroid immune status needs further study.

